# Factorial Network Models to Improve P2P Credit Risk Management

**DOI:** 10.3389/frai.2019.00008

**Published:** 2019-06-04

**Authors:** Daniel Felix Ahelegbey, Paolo Giudici, Branka Hadji-Misheva

**Affiliations:** ^1^Department of Mathematics and Statistics, Boston University, Boston, MA, United States; ^2^Department of Economics and Management, University of Pavia, Pavia, Italy; ^3^Zurich University of Applied Sciences (ZHAW) University of Applied Sciences, Zurich, Switzerland

**Keywords:** credit risk, factor models, FinTech, peer-to-peer lending, credit scoring, lasso, segmentation

## Abstract

This paper investigates how to improve statistical-based credit scoring of SMEs involved in P2P lending. The methodology discussed in the paper is a factor network-based segmentation for credit score modeling. The approach first constructs a network of SMEs where links emerge from comovement of latent factors, which allows us to segment the heterogeneous population into clusters. We then build a credit score model for each cluster via lasso-type regularization logistic regression. We compare our approach with the conventional logistic model by analyzing the credit score of over 1,5000 SMEs engaged in P2P lending services across Europe. The result reveals that credit risk modeling using our network-based segmentation achieves higher predictive performance than the conventional model.

## 1. Introduction

Issuance of loans by traditional financial institutions, such as banks, to other firms and individuals, is often associated with major risks. The failure of loan recipients to honor their obligation at the time of maturity leaves the banks vulnerable and affects their operations. The risk associated with such transactions is referred to as credit risk. It is well known that some percentage of these non-performing loans are eventually imputed to economic losses. To minimize such risk exposures, various methods have been extensively discussed in the credit risk literature to enable credit-issuing institutions to undertake a thorough assessment to classify loan applicants into risky and non-risky customers. Some of these methods range from logistic and linear probability models to decision trees, neural networks and support vector machines. A conventional individual-level reduced-form approach is the credit scoring model which attributes a score of credit-worthiness to each loan applicant based on the available history of their financial characteristics. See Altman (1968) for some pioneer works on corporate bankruptcy prediction models using accounting-based measures as variables. For a comprehensive review on credit scoring models, see Alam et al. (2010).

Recent advancements gradually transforming the traditional economic and financial system is the emergence of digital-based systems. Such systems present a paradigm shift from traditional infrastructural systems to technological (digital) systems. Financial technological (“FinTech”) companies are gradually gaining ground in major developed economies across the world. The emergence of Peer-to-Peer (P2P) platforms is a typical example of a FinTech system. The P2P platform aims at facilitating credit services by connecting individual lenders with individual borrowers without the interference of traditional banks as intermediaries. Such platform serves as a digital financial market and an alternative to the traditional physical financial market. P2P platforms significantly improve the customer experience and the speed of the service and reduce costs to both individual borrowers and lenders as well as small business owners. Despite the various advantages, P2P systems inherit some of the challenges of traditional credit risk management. In addition, they are characterized by the asymmetry of information and by a strong interconnectedness among their users (see e.g., Giudici et al., 2019) that makes distinguishing healthy and risky credit applicants difficult, thus affecting credit issuers. There is, therefore, a need to explore methods that can help improve credit scoring of individual or companies that engage in P2P credit services.

This paper investigates how factor-network-based segmentation can be employed to improve the statistical-based credit score for small and medium enterprises (SMEs) involved in P2P lending. The approach is to first constructs a network of SMEs where links emerge from comovement of the latent factors that drive the observed financial characteristics. The network structure then allows us to segment the heterogeneous population into two sub-groups of connected and non-connected clusters. We then build a credit score model for each sub-population via lasso-type regularization logistic regression.

The contribution to the literature of this paper is manifold. Firstly, we extend the ideas contained in the factor network-based classification of Ahelegbey et al. (2019) to a more realistic setting, characterized by a large number of observations which, when links between them are the main object of analysis, becomes extremely challenging.

Secondly, we extend the network-based scoring model proposed in Giudici et al. (2019) to a setting characterized by a large number of explanatory variables. The variables are selected via lasso-type regularization (Tibshirani, 1996; Hastie et al., 2009) and, then, summarized by factor scores. Thus, we contribute to network-based models for credit risk quantification. Network models have been shown to be effective in gauging the vulnerabilities among financial institutions for risk transmission (see Battiston et al., 2012; Billio et al., 2012; Diebold and Yilmaz, 2014; Ahelegbey et al., 2016a), and a scheme to complement micro-prudential supervision with macro-prudential surveillance to ensure financial stability (see IMF, 2011; Moghadam and Viñals, 2010; Viñals et al., 2012). Recent application of networks have been shown to improve loan default predictions and capturing information that reflects underlying common features (see Letizia and Lillo, 2018; Ahelegbey et al., 2019).

Thirdly, our empirical application contributes to modeling credit risk in SMEs particularly engaged in P2P lending. For related works on P2P lending via logistic regression (see Andreeva et al., 2007; Barrios et al., 2014; Emekter et al., 2015; Serrano-Cinca and Gutiérrez-Nieto, 2016). We model the credit score of over 15,000 SMEs engaged in P2P credit services across Southern Europe. We compare the performance of our network-based segmentation credit score model (NS-CSM) with the conventional single credit score model (CSM). We show via our empirical results that our network-based segmentation presents a more efficient scheme that achieves higher performance than the conventional approach.

The paper is organized as follows. Section 2 presents the factor network segmentation methodology and the lasso-type regularization for credit scoring. Section 3 discusses the empirical application of our segmentation approach against the conventional single model.

## 2. Methodology

We present the formulation and inference of a latent factor network to improve credit scoring and model estimation. Our objective is to analyze the characteristics of the borrowers to build a model that predicts the likelihood of their default.

### 2.1. Logistic Model

Let *Y* be a vector of independent observations of the loan status of *n* firms, such that *Y*_*i*_ = 1 if firm-*i* has defaulted on its loan obligation, and zero otherwise. Furthermore, let *X* = {*X*_*ij*_}, *i* = 1, …, *n, j* = 1, …, *p*, be a matrix of *n* observations with *p* financial characteristic variables or predictors. The conventional parameterization of the conditional distribution of *Y* given *X* is the logistic model with log-odds ratio given by

(1) $$\begin{array}{l} \log\left(\frac{\pi_{i}}{1-\pi_{i}}\right)=\beta_{0}+X_{i}\beta \end{array}$$

where π_*i*_ = *P*(*Y*_*i*_ = 1|*X*_*i*_), β_0_ is a constant term, $\;\beta ={\left(\beta_{1},\ldots ,\beta_{p}\right)}^{\prime }$ is a *p* × 1 vector of coefficients and *X*_*i*_ is the *i*-th row of *X*.

### 2.2. Decomposition of Data Matrix by Factors

The dataset *X* can be considered as points of *n*-institutions in a *p*-dimensional space. It can also be interpreted at observed outcomes driven by some underlying firm characteristics. More specifically, *X* can be expressed as a factor model given by

(2) $$\begin{array}{l} X\;=\;FW+\varepsilon \end{array}$$

where *F* is *n*×*k* matrix of latent factors, *W* is *p*×*k* matrix of factor loadings, ε is *n* × *p* matrix of errors uncorrelated with *F*. The error term ε is typically assumed to be multivariate normal but *F* in general case need not be multivariate normal (see Tabachnick et al., 2007). Lastly, *k* < *p* is the number of factors required to summarize the pattern of correlations in the observed data matrix *X*. In the context of our application, we set *k* to be the number of factors that account for approximately 95% of the variation in *X*.

### 2.3. Factor Network-Based Segmentation

We present the construction of network structure for the segmentation of the population. Following the literature on graphical models (see Carvalho and West, 2007; Eichler, 2007; Ahelegbey et al., 2016a,b), we represent the network structure as an undirected binary matrix, *G* ∈ {0, 1}^*n*×*n*^, where *G*_*ij*_ represents the presence or absence of a link between nodes *i* and *j*. We construct *G* via similarity of the latent firm characteristics, such that *G*_*ij*_ = 1 if the latent coordinates of firm-*i* are strongly related to firm-*j*, and zero otherwise.

Given the latent factors matrix, *F*, we construct a network where the marginal probability of a link between nodes-*i* and *j* by

(3) $$\begin{array}{l} \gamma_{ij}\;=\;P\left(G_{ij}=1|F\right)\;=\;\Phi \left[\theta +{\left(FF^{\prime }\right)}_{ij}\right] \end{array}$$

where γ_*ij*_ ∈ (0, 1), Φ is the standard normal cumulative density function, θ ∈ ℝ is a network density parameter, and ${\left(FF^{\prime }\right)}_{ij}$ is the *i*-th row and the *j*-th column of *FF*′. Under the assumption that *G* is undirected, it follows that γ_*ij*_ = *P*(*G*_*ij*_ = 1|*F*) = *P*(*G*_*ji*_ = 1|*F*) = γ_*ji*_. We validate the link between nodes-*i* and *j* in *G* by

(4) $$\begin{array}{ll} G_{ij}\; & =\;1\left(\gamma_{ij}>\gamma \right) \end{array}$$

where **1**(γ_*ij*_ > γ) is the indicator function, i.e., unity if γ_*ij*_ > γ and zero otherwise, and γ ∈ (0, 1) is a threshold parameter. By definition, the parameters θ and γ control the density of *G*. Following Ahelegbey et al. (2019), we set $\theta =\Phi^{-1}\left(\frac{2}{n-1}\right)$. To broaden the robustness of the results, we compare γ = {0.05, 0.1} to capture a sparse but closely connected community.

### 2.4. Estimating High-Dimensional Logistic Models

When estimating high-dimensional logistic models with a relatively large number of predictors, there is the tendency to have redundant explanatory variables. Thus, to construct a predictable model, there is the need to select the subset of predictors that explains a large variation in the probability of defaults. Several variable selection methods have been discussed and applied for various regression models. In this paper, we consider variants of the lasso regularization for logistic regressions (Hastie et al., 2009).

#### 2.4.1. Lasso

The lasso estimator (Tibshirani, 1996) solves a penalized log-likelihood function given by

(5) $$\begin{array}{l} \arg\;\underset{\beta }{\min}\sum_{i=1}^{n}[Y_{i}(\beta_{0}+X_{i}\beta )-\log\left(1+\exp(\beta_{0}+X_{i}\beta )\right)]\\ -\lambda \sum_{j=0}^{p}|\beta_{j}| \end{array}$$

where *n* is the number of observations, *p* the number of predictors, and λ is the penalty term, such that large values of λ shrinks a large number of the coefficients toward zero.

#### 2.4.2. Adaptive Lasso

The adaptive lasso estimator (Zou, 2006) is an extension of the lasso that solves

(6) $$\begin{array}{l} \arg\;\underset{\beta }{\min}\sum_{i=1}^{n}[Y_{i}(\beta_{0}+X_{i}\beta )-\log\left(1+\exp(\beta_{0}+X_{i}\beta )\right)]\\ -\lambda \sum_{j=0}^{p}w_{j}|\beta_{j}| \end{array}$$

where *w*_*j*_ is a weight penalty such that $w_{j}=1/|{\hat{\beta }}_{j}{|}^{v}$, with ${\hat{\beta }}_{j}$ as the ordinary least squares (or ridge regression) estimate and *v* > 0.

#### 2.4.3. Elastic-Net

The elastic-net estimator (Zou and Hastie, 2005) solves the following

(7) $$\begin{array}{l} \arg\;\underset{\beta }{\min}\sum_{i=1}^{n}[Y_{i}(\beta_{0}+X_{i}\beta )-\log\left(1+\exp(\beta_{0}+X_{i}\beta )\right)]\\ -\lambda \sum_{j=0}^{p}(\alpha |\beta_{j}|+(1-\alpha )\beta_{j}^{2}) \end{array}$$

where α ∈ (0, 1) is an additional penalty such that when α = 1 we a lasso estimator (*L*_1_ penalty), and when α = 0 a ridge estimator (*L*_2_ penalty). For the elastic-net estimator, we set α = 0.5 giving equal weight to the *L*_1_ and *L*_2_ regularization.

#### 2.4.4. Adaptive Elastic-Net

The adaptive elastic-net estimator (Zou and Zhang, 2009) combines the additional penalties of the adaptive lasso and the elastic-net to solve the following

(8) $$\begin{array}{l} \arg\;\underset{\beta }{\min}\sum_{i=1}^{n}[Y_{i}(\beta_{0}+X_{i}\beta )-\log\left(1+\exp(\beta_{0}+X_{i}\beta )\right)]\\ -\lambda \sum_{j=0}^{p}(\alpha w_{j}|\beta_{j}|+(1-\alpha )\beta_{j}^{2}) \end{array}$$

In the empirical work, we focus on estimating the credit score using the four lasso-type regularization methods. We select the regularization parameter using 10-fold cross-validation on a grid of λ values for the penalized logistic regression problem. Two λ's are widely considered in the literature, i.e., λ.*min* and λ.1*se*. The former is the value of the λ that minimizes the mean square cross-validated errors, while the latter is the λ value that corresponds to one standard error from the minimum mean square cross-validated errors. Our preliminary analysis shows that λ.1*se* produces a larger penalty that is too restrictive in the sense that we lose almost all the regressors. Although our goal is to encourage a sparse credit scoring model for the purpose of interpretability, we do not want to impose too much sparsity that renders the majority of the features insignificant. Thus, we rather choose λ.*min* over λ.1*se*. For the additional penalty terms, we set α = 0.5, *v* = 2, and ${\hat{\beta }}_{j}$ as the ridge regression estimate.

## 3. Application

### 3.1. Data: Description and Summary Statistics

To illustrate the effectiveness of the application of factor network methodology in credit scoring analysis, we obtained data from the European External Credit Assessment Institution (ECAI) on 15045 small-medium enterprises engaged in Peer-to-Peer lending on digital platforms across Southern Europe.

The observation on each institution is composed of 24 financial characteristic ratios constructed from official financial information recorded in 2015. Table 1 presents a description of the financial ratios with summary of mean statistics of the institutions grouped according to their default status. In all, the data consists of 1,632 (10.85%) defaulted institutions and 13,413 (89.15%) non-defaulted companies.

**Table 1:** Description of the financial ratios with summary of mean statistics according to default status.

**Var**	**Formula (description)**	**Active (mean)**	**Defaulted (mean)**
V1	(Total Assets - Shareholders Funds)/Shareholders Funds	8.87	9.08
V2	(Longterm debt + Loans)/Shareholders Funds	1.25	1.32
V3	Total Assets/Total Liabilities	1.51	1.07
V4	Current Assets/Current Liabilities	1.6	1.06
V5	(Current Assets - Current assets: stocks)/Current Liabilities	1.24	0.79
V6	(Shareholders Funds + Non current liabilities)/Fixed Assets	8.07	5.99
V7	EBIT/Interest paid	26.39	−2.75
V8	(Profit (loss) before tax + Interest paid)/Total Assets	0.05	−0.13
V9	P/L after tax/Shareholders Funds	0.02	−0.73
V10	Operating Revenues/Total Assets	1.38	1.27
V11	Sales/Total Assets	1.34	1.25
V12	Interest Paid/(Profit before taxes + Interest Paid)	0.21	0.08
V13	EBITDA/Interest Paid	40.91	5.71
V14	EBITDA/Operating Revenues	0.08	−0.12
V15	EBITDA/Sales	0.09	−0.12
V16	Constraint EBIT	0.13	0.56
V17	Constraint PL before tax	0.16	0.61
V18	Constraint Financial PL	0.93	0.98
V19	Constraint P/L for period	0.19	0.64
V20	Trade Payables/Operating Revenues	100.3	139.30
V21	Trade Receivables/Operating Revenues	67.59	147.12
V22	Inventories/Operating Revenues	90.99	134.93
V23	Total Revenue	3557	2083
V24	Industry Classification on NACE code	4566	4624
	Total number of institutions (%)	13413(89.15%)	1632(10.85%)

### 3.2. Decomposition of the Observed Data Matrix by Factors

To estimate the underlying factors that drive the observed data matrix, we decompose the matrix of observed financial characteristics via a singular value decomposition given by,

(9) $$\begin{array}{l} X\;=\;UDV\;=\;FW+\varepsilon \end{array}$$

where *U* and *V* are orthonormal, and *D* = Λ^1/2^ is a diagonal matrix of non-negative and decreasing singular values, with Λ as the diagonal matrix of the non-zero eigenvalues of *X*′*X* and *XX*′. *U* is *n* × *p*, *D* is *p* × *p* and *V* is *p* × *p*. Following the error approximation criteria, we obtain the factor matrix by, *F* = *U*_*n,k*_*D*_*k,k*_ and *W* = *V*_*k,p*_, where *U*_*n,k*_ is *n* × *k* matrix composed of the first *k* columns of *U*, *k* < *p*, *D*_*k,k*_ is *k*×*k* matrix comprising the first *k* columns and rows of *D*, and *V*_*k,p*_ is *k* × *p* matrix of factor loadings. The matrix *F* can therefore be interpreted as a projection of *X* onto the eigenspace spanned by *U*_*n,k*_. We determine *k* by observing the number of eigenvalues associated with the largest variance matrix. Table 2 shows the eigenvalues of the singular value decomposition to determine the factors to retain. The eigenvalues reported are the normalized squared diagonal terms of *D*. From the table, we set *k* = 17 since the first 17 eigenvalues explain about 95% of the total variation in *X*.

**Table 2:** The eigenvalues of the singular value decomposition to determine the factors to retain.

**No**.	**Eigenvalue**	**Variance explained (%)**	**Cumulative (%)**
1	5.18	21.60	21.60
2	2.58	10.73	32.33
3	2.50	10.41	42.74
4	1.60	6.69	49.42
5	1.42	5.92	55.34
6	1.30	5.40	60.74
7	1.16	4.82	65.55
8	1.09	4.56	70.11
9	0.99	4.11	74.22
10	0.93	3.88	78.10
11	0.80	3.35	81.45
12	0.79	3.31	84.76
13	0.75	3.11	87.87
14	0.56	2.35	90.22
15	0.53	2.21	92.43
16	0.51	2.12	94.55
17	0.43	1.80	96.35
18	0.37	1.54	97.89
19	0.17	0.69	98.58
20	0.11	0.47	99.05
21	0.09	0.36	99.41
22	0.07	0.27	99.68
23	0.06	0.26	99.94
24	0.01	0.06	100.00

### 3.3. Factor Network Analysis

**Figure 1:**
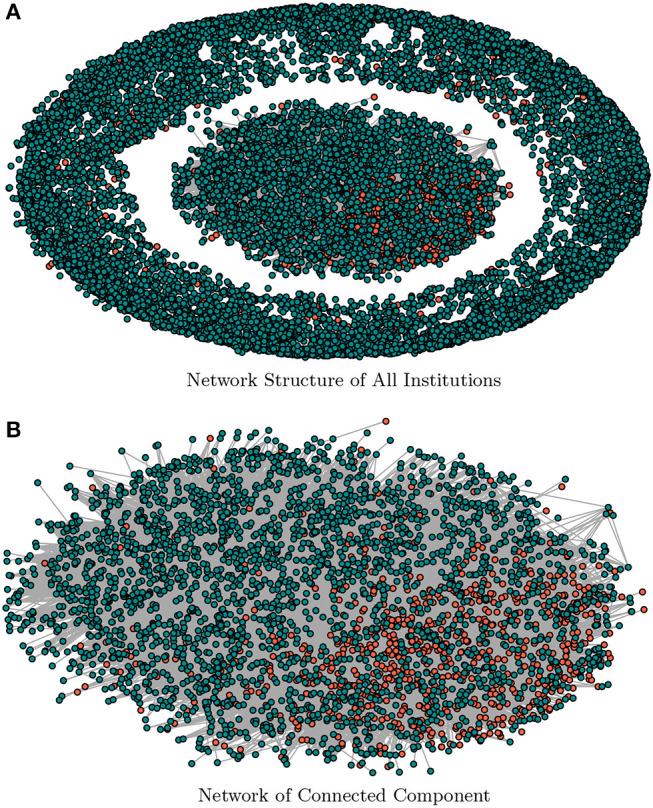
A graphical representation of the estimated factor network. **(A)** shows the structural representation of the factor network for threshold γ = 0.05, and **(B)** depicts the connected sub-population only. The nodes in red-color are defaulted class of companies and green-color coded nodes are non-defaulted class of companies. **(A)** Network Structure of All Institutions. **(B)** Network of Connected Component.

We use the estimated factor matrix, *F*, to construct the network for the segmentation of the companies. For purposes of graphical representations and to keep the companies name anonymous, we report the estimated network by representing the group of institutions with color-codes. The defaulted companies are represented in a red color code, and non-defaulted companies in the green color code (see Figure 1). Table 3 reports the summary statistics of the estimated network in terms of the default-status composition of the SMEs. For robustness purposes, we compare the results obtained with a threshold value γ = 0.05 against γ = 0.10.

**Table 3:** Summary statistic of connected and non-connected sub-population obtained from the factor network-based segmentation for threshold values of γ = {0.05, 0.1}.

**Threshold**	**Status**	**Conn-sub**		**Non-conn-sub**	
γ = 0.05	Default	964	22.4%	668	6.2%
	Non-Default	3,341	77.6%	10,072	93.8%
	Total	4,305	28.6%	10,740	71.4%
γ = 0.1	Default	816	24%	816	7%
	Non-Default	2,580	76%	10,833	93%
	Total	3,396	22.6%	11,649	77.6%

The result for the threshold γ = 0.05 of Table 3 shows that the connected sub-population is composed of 4,305 companies which constitute 28.6% of the full sample. The non-connected sub-population is composed of 10,740 (71.4%). The percentage of the defaulted class of companies are 22.4 and 6.2% among the connected- and non-connected sub-population, respectively. We notice that higher threshold values (say γ = 0.1) decrease (increase) the total number of connected (non-connected) sub-population and vice versa. Such higher threshold values also lead to a lower (higher) number of defaulted class of connected (non-connected) SMEs but (and) constituting a higher percentage of the defaulted population. Figure 1 presents the graphical representation of the estimated factor network with the sub-population of defaulted and non-defaulted companies color coded as red and green, respectively. Figure 1A shows the structural representation of both connected and non-connected sub-population while Figure 1B depicts the structure of connected sub-population only.

### 3.4. Credit Score Modeling

We compare the lasso, adaptive lasso, elastic-net, and adaptive elastic-net variable selection methods to model the credit score of the listed companies in our dataset. To estimate the models, we standardized each series to a zero mean and unit variance. Table 4 reports the variable selection and estimated coefficients of the four methods. The column CSM represents the benchmark credit scoring model, NS-CSM(C) - the network segmented connected sub-population credit scoring model, and NS-CSM(NC) for the network segmented non-connected sub-population credit scoring model. The top left panel represents the lasso method, the adaptive lasso is on the top right panel, elastic-net at the bottom left and adaptive elastic-net at the bottom right.

**Table 4:** Estimated coefficients from lasso (top left), adaptive lasso (top right), elastic-net (bottom left) and adaptive elastic-net (bottom right).

	**CSM**	**NS-CSM(C)**	**NS-CSM(NC)**	**CSM**	**NS-CSM(C)**	**NS-CSM(NC)**
	**lasso**			**Adaptive lasso**		
V1	0.0535	·	0.0375	·	·	·
V2	·	0.0332	·	·	·	·
V3	−0.4468	−0.2818	−1.0148	−0.5298	−0.3539	−1.1990
V4	−0.3549	−0.1294	−0.5556	−0.2928	−0.1368	−0.5137
V5	·	·	·	·	·	·
V6	0.0774	·	0.1460	0.0440	·	0.0213
V7	0.2818	·	·	0.2116	·	·
V8	−0.3933	−0.3408	0.1185	−0.4356	−0.3463	·
V9	−0.0360	0.0365	−0.4690	·	·	−0.5577
V10	−0.0701	0.0287	·	·	·	·
V11	0.1291	·	0.0550	·	·	·
V12	0.0265	0.0222	0.0204	·	·	·
V13	−0.2419	·	·	−0.1759	·	·
V14	−0.0399	−0.0776	·	·	−0.113	·
V15	−0.0751	−0.0396	0.0128	−0.0520	·	·
V16	0.0520	0.2851	·	·	0.2245	·
V17	0.2213	0.1650	0.1761	0.2529	0.2092	·
V18	0.0396	0.0661	0.0143	·	0.0484	·
V19	0.2540	0.0291	0.2096	0.2755	·	0.2151
V20	0.0412	·	0.2429	·	·	0.1950
V21	0.2212	0.1620	0.2969	0.2410	0.1721	0.3185
V22	0.0930	·	0.1470	0.0541	·	0.0219
V23	−0.2262	−0.0649	−0.3452	−0.2213	−0.0650	−0.3826
V24	−0.0062	−0.0641	0.0343	·	−0.0645	·
	**Elastic-net**			**Adaptive elastic-net**		
V1	0.0548	·	0.0568	·	·	·
V2	1.0*e*−04	0.0372	·	·	·	·
V3	−0.4472	−0.2692	−1.0132	−0.5293	−0.3538	−1.2208
V4	−0.3628	−0.1286	−0.6051	−0.2900	−0.1350	−0.6034
V5	0.0048	−0.0123	·	·	·	·
V6	0.0780	−0.0028	0.1862	0.0422	·	0.1528
V7	0.3003	·	·	0.1925	·	·
V8	−0.3926	−0.3310	0.2054	−0.4363	−0.3474	0.1672
V9	−0.0356	0.0435	−0.4884	·	·	−0.5195
V10	−0.1419	0.0315	·	·	·	·
V11	0.2016	0.0112	0.1025	·	·	·
V12	0.0299	0.0299	0.0545	·	·	·
V13	−0.2595	·	·	−0.1571	·	·
V14	−0.0374	−0.0785	·	·	−0.1112	·
V15	−0.0777	−0.0468	0.0597	−0.0499	·	·
V16	0.0600	0.2902	0.0669	·	0.2256	·
V17	0.2173	0.1588	0.1701	0.2527	0.2097	0.1147
V18	0.0417	0.0769	0.0439	·	0.0459	·
V19	0.2538	0.0502	0.2042	0.2747	·	0.2151
V20	0.0425	·	0.3139	·	·	0.2571
V21	0.2210	0.1634	0.3113	0.2409	0.1721	0.3036
V22	0.0933	0.0012	0.1727	0.0533	·	0.1047
V23	−0.2286	−0.0728	−0.3754	−0.2185	−0.0616	−0.4114
V24	−0.0077	−0.0724	0.0464	·	−0.0619	·

*CSM is the benchmark credit score model, NS-CSM(C) is the network segmented connected sub-population credit score model, and NS-CSM(NC) is the network segmented non-connected sub-population credit score model, estimated for threshold value γ = 0.1*.

Table 5 reports the number of variables selected by each of the four competing methods for the credit score model estimation. From the table, the elastic-net is the least parsimonious, followed by the lasso, and lastly, the adaptive elastic-net and adaptive lasso are the most parsimonious. From Tables 4, 5, we observed a significant difference in the number of selected explanatory variables for the benchmark model and the network segmented models. More precisely, the former model the credit score of a given company by using more variables while the latter on the other hand uses a significantly lower number of variables. The similar results across the four variable selection methods, given their similarities, is not terribly surprising. But they do indicate that the general approach appears to be robust in this setting, which was the main purpose of the testing. The network-based segmentation framework is therefore more parsimonious than the benchmark full population credit score model, and this helps in interpretability.

**Table 5:** Number of selected variables of the four methods.

	**Lasso**	**Adaptive lasso**	**Elastic-net**	**Adaptive elastic-net**
CSM	22	12	24	12
NS-CSM(C)	16	10	20	10
NS-CSM(NC)	17	9	18	11

### 3.5. Comparing Default Predicting Accuracy

**Figure 2:**
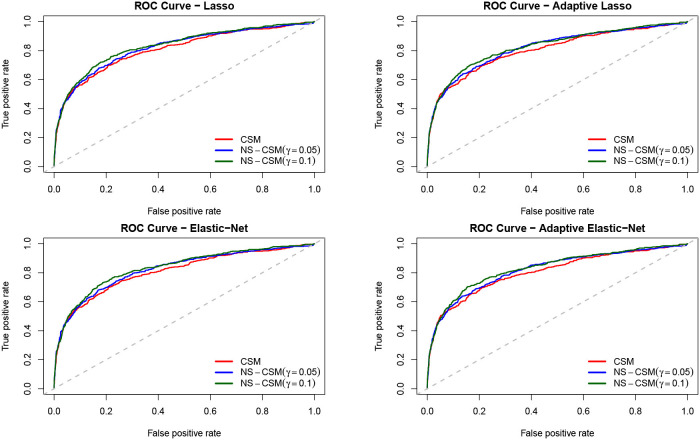
ROC curves of the four methods. CSM is the benchmark model, NS-CSM(C) is the network segmented connected sub-population model, and NS-CSM(NC) is the network segmented non-connected sub-population model, estimated for threshold values of γ = {0.05, 0.1}.

We analyzed the performance of the models by splitting the sample into 70% training and 30% testing sample. We now compare the default prediction accuracy of the models in terms of the standard area under the curve (AUC) derived from the receiver operator characteristic (ROC) curve. The AUC depicts the true positive rate (TPR) against the false positive rate (FPR) depending on some threshold. TPR is the number of correct positive predictions divided by the total number of positives. FPR is the ratio of false positives predictions overall negatives. See Figure 2 for the plot of the ROC curve for the competing methods.

The comparison of the ROC curves from the competing methods shows that the CSM (in red) lies below the rest. Clearly, the curves of NS-CSM (γ = 0.1) depicted in green seems to dominate the others. The summary of the area under the ROC curve reported in Table 6 shows that NS-CSM (γ = 0.1) is ranked first, followed by NS-CSM (γ = 0.05), and the lowest AUC is obtained by the CSM. Overall, in terms of default predictive accuracy, the result of the AUC shows the NS-CSM outperforms the CSM, on average by two percentage points. This is an advantage that can be further increased considering as the cut-off the observed default percentages, which are different in the two samples.

**Table 6:** Comparing area under the ROC curve (AUC) of the four methods.

	**Lasso**	**Adaptive lasso**	**Elastic-net**	**Adaptive elastic-net**
CSM	0.8089	0.8061	0.8090	0.8061
NS-CSM(γ = 0.05)	0.8214	0.8204	0.8225	0.8207
NS-CSM(γ = 0.1)	0.8330	0.8277	0.8342	0.8312

We investigate whether the AUC of the network segmented model is significantly different from the benchmark model for the four methods. We applied the DeLong test (DeLong et al., 1988) to investigate the pairwise comparison of the AUC of the benchmark model (i.e., CSM) and that of the NS-CSM for γ = {0.05, 0.1}. We perform these tests under the null-hypotheses that *H*_0_: AUC (CSM) ≥ AUC (NS-CSM) and the alternative hypotheses, *H*_1_: AUC (CSM) < AUC (NS-CSM). Table 7 reports the one-sided statistical test of the AUC of the benchmark model relative to the network segmented models. The result of the De Long test shows that while the ROC of CSM is not statistically different from that of NS-CSM(γ = 0.05), the difference between the ROC of NS-CSM(γ = 0.1) and the benchmark (CSM) is statistically significant at 90% confidence level for all four methods.

**Table 7:** AUC of the benchmark model relative to the network segmented models under the four methods.

		**Statistic**	***P*****-value**	**Significance**	**Statistic**	***P*****-value**	**Significance**
		Lasso			Adaptive lasso		
CSM	NS-CSM(γ = 0.05)	-0.7639	0.2225		-0.8598	0.1950	
	NS-CSM(γ = 0.1)	-1.4972	0.0672	^*^	-1.3129	0.0946	^*^
		Elastic-net			Adaptive elastic-net		
CSM	NS-CSM(γ = 0.05)	-0.8241	0.2050		-0.8728	0.1914	
	NS-CSM(γ = 0.1)	-1.5770	0.0574	^*^	-1.5327	0.0627	^*^

In conclusion, our proposed factor network approach to credit score modeling presents an efficient framework to analyze the interconnections among the borrowers of a peer to peer platform and provides a way to segment a heterogeneous population into clusters with more homogeneous characteristics. The results show that the lasso logistic model for credit scoring leads to better identification of the significant set of relevant financial characteristic variables, thereby producing a more interpretable model, especially when combined with the segmentation of the population via the factor network-based approach. These empirical results are promising, but certainly not definitive. More research is required to determine whether the observed ‘lift’ truly is significant rather than just an artifact of random chance or spurious correlation, especially given the fact that these *p*-values are not calibrated in any way (e.g., Sellke et al., 2001) and Calabrese and Giudici (2015). Further research may include a Bayesian approach, as in Figini and Giudici (2011) and Giudici (2001). We therefore find evidence of a modest improvement in the default predictive performance of our model compared to the conventional approach.

## 4. Conclusion

This paper improves credit risk management of SMEs engaged in P2P credit services by proposing a factor network-based approach to segment a heterogeneous population into a cluster of homogeneous sub-populations and estimating a credit score model on the clusters using a lasso-type regularization logistic model.

We demonstrate the effectiveness of our approach through empirical applications analyzing the probability of default of over 15,000 SMEs involved in P2P lending across Europe. We compare the results from our model with the one obtained with standard single credit score methods. We find evidence that our factor network approach helps to obtain sub-population clusters such that the resulting models associated with these clusters are more parsimonious than the conventional full population approach, leading to better interpretability and to a modest improved default predictive performance.

## Data Availability

All datasets generated for this study are included in the manuscript and/or the supplementary files.

## Author Contributions

In this manuscript, all the authors investigated how to improve the credit scoring of SMEs involved in P2P lending via a factor network-based segmentation method. The contribution of this work is manifold. DA extended a recently proposed concept of factor network-based classification to a more realistic setting. PG contributed to network-based models for credit risk quantification using a lasso logistic regression. BH-M presented an application of our approach to model the credit score of over 15,000 SMEs engaged in P2P credit services across Southern Europe.

### Conflict of Interest Statement

The authors declare that the research was conducted in the absence of any commercial or financial relationships that could be construed as a potential conflict of interest.
